# Paraquat Reduces the Female Fertility by Impairing the Oocyte Maturation in Mice

**DOI:** 10.3389/fcell.2020.631104

**Published:** 2021-02-02

**Authors:** Yan-Li Sun, Xue-Lin Wang, Lei-Lei Yang, Zhao-Jia Ge, Yong Zhao, Shi-Ming Luo, Wei Shen, Qing-Yuan Sun, Shen Yin

**Affiliations:** ^1^College of Animal Science and Technology, Qingdao Agricultural University, Qingdao, China; ^2^College of Life Sciences, Institute of Reproductive Science, Key Laboratory of Animal Reproduction and Germplasm Enhancement in Universities of Shandong, Qingdao Agricultural University, Qingdao, China; ^3^College of Science, Health, Engineering and Education, Murdoch University, Murdoch, WA, Australia; ^4^Fertility Preservation Lab, Reproductive Medicine Center, Guangdong Second Provincial General Hospital, Guangzhou, China

**Keywords:** paraquat, oocyte, fertility, kinetochore-microtubule attachment, epigenetic modification

## Abstract

Paraquat (PQ) is a widely used non-selective and oxidizing herbicide in farmland, orchards, flower nursery, and grassland. Overuse of PQ will accumulate in the body and affect the reproduction in mammals. In this study, we found that PQ could reduce the female fertility by oral administration for 21 days in mice. PQ exposure could impair the nuclear maturation by perturbing the spindle assembly and kinetochore–microtubule attachment to cause the misaligned chromosomes during meiosis. In the meantime, PQ exposure disturbed the mitochondrial distribution and enhanced the level of reactive oxygen species and early apoptosis, which thereby deteriorated the early embryo development. Also, PQ administration could cause some changes in epigenetic modifications such as the level of H3K9me2 and H3K27me3. Therefore, PQ administration reduces the female fertility by impairing the nuclear and cytoplasmic maturation of oocytes in mice.

## Introduction

More and more herbicides are widely used in farmland, orchards, flower nursery, and grassland, especially in developing countries. Paraquat (PQ; 1,1’-dimethyl-4,4’-bipyridinium dichloride) is an effectively and widely non-selective, water-soluble herbicide. It is extremely toxic to humans and animals in many aspects (Kumar et al., 2016). The number of patients worldwide who had been poisoned by PQ was over 650,000 in 2016 (Nunes et al., 2017), and it is predicted to exceed 1 million by 2025 (Ding et al., 2016).

Many researches on PQ were focused on its neurotoxicity. PQ can affect the proliferation and differentiation of neural stem cells and the expression of neuronal differentiation-related genes (Huang et al., 2019). Moreover, PQ exposure increases the mitochondrial fragmentation and alters the DNA methylation in human neural stem cells (Xiong et al., 2019). For reproductive research, PQ can transform the hypothalamic–pituitary–gonadal (HPG) axis to affect the reproductive functions in Japanese quails (Soni et al., 2019). Male rats were given PQ by oral administration for a short time, which could retard the regeneration of Leydig cells from stem/progenitor Leydig cells, decrease the production of testosterone, and block the spermatogenesis (Li et al., 2019). In addition, PQ can reduce the *in vitro* fertilization (IVF) outcomes from oxidative stress (Pang et al., 2019; Kamali et al., 2020).

In female, the quality of mature oocyte plays a decisive role in early embryonic development and fertility (Lonergan and Fair, 2016). Before meiotic maturation, oocytes are arrested at the prophase of meiosis I with a distinct germinal vesicle (GV). In puberty, fully grown oocytes reinitiate meiosis and complete the first meiotic division with the first polar body (PB1) extrusion and then arrest at the metaphase of meiosis II (MII) waiting for fertilization (Downs, 2015). The meiotic maturation of oocyte is a long and complex process, which is divided into nuclear and cytoplasmic maturation for simplicity (Marteil et al., 2009). The nuclear maturation includes all the events related to the faithful chromosome alignment and segregation. The cytoplasmic maturation involves the organelle maturation and redistribution, as well as the storage of important molecules. For example, mitochondria localize at the perinuclear area in the GV stage and then shift to the inner cytoplasm during meiotic progression (Sun et al., 2001). The substantial storage of mRNA, lipids, proteins, and other transcriptional factors in the cytoplasm plays vital roles during oocyte maturation, fertilization, and early embryo development (Ferreira et al., 2009; Duan et al., 2015). In addition, epigenetic changes during oocyte maturation (epigenetic maturation) including specific histone methylation pattern are related to early embryo development; some adverse effects can be passed to the next generation by epigenetics (Hirota et al., 2005; Govin et al., 2010).

In this study, we evaluated the toxic effects of PQ on the reproduction of female mice by oral administration. The results showed that PQ administration could decrease the quality of oocyte to weaken the female fertility by impairing the nuclear and cytoplasmic maturation during meiotic progression. Also, PQ exposure caused some changes in the epigenetic modifications. All these results provide a new insight into the reproductive toxicity of PQ *in vivo*.

## Materials and Methods

### Animal Husbandry

All the animal studies were approved by the Animal Research Committee of Qingdao Agricultural University. Three-week-old CD-1 female mice were raised in a temperature-controlled room with 12-h day/dark cycling and regularly given a normal diet and water. Pain relief was considered, and the mice were humanely treated as much as possible.

### Chemical Administration

Female mice were randomly divided into two groups (18–20/group). PQ (Aladdin, Shanghai, China) was dissolved in saline at a stock concentration of 120 mg/ml. Female mice were weighed by using an S-234 scale at 4:00 p.m. every other day. PQ was given to female mice in a dose of 10 mg/kg average body weight every day by oral administration for 21 days, and the control group was given saline according to the previous studies (Krishna et al., 2019; Soni et al., 2019).

### Oocyte Collection and Maturation Culture

Denuded oocytes with a distinct GV were collected from the ovaries of control and PQ-treated mice in M2 medium with 2.5 μM of milrinone (MedChemExpress, New Jersey, United States) (Yang et al., 2019). After washing, oocytes were cultured in the 35 μl drops of M16 (Sigma-Aldrich, MO, United States) under mineral oil for *in vitro* maturation (8 or 12 h to MI or MII stage) in a condition of 5% CO_2_ atmosphere at 37°C. Each group has 25–30 oocytes.

### Early Embryo Collection and Culture

Female mice were regularly mated with male mice at 5:00–5:30 p.m., and vaginal plug was checked at 8:30–9:00 a.m. on the next morning. Oviductal ampullae were broken by syringe to release the cumulus oocyte complexes (COCs). Cumulus cells of COCs were removed in M2 medium supplement with 0.1% hyaluronidase (Sigma-Aldrich) to get cumulus-free oocytes and transferred to KSOM (EMD Millipore Corp, Billerica, MA, United States) to culture until blastocyst stage (Sun et al., 2019). IVF was conducted with the standard procedure as before (Yang et al., 2019).

### Immunofluorescent Staining

Oocytes at MI stage were observed with the standard procedure as published before (Yang et al., 2019). The primary antibodies included mouse monoclonal anti-α-tubulin fluorescein isothiocyanate (FITC) antibody (1:500, Santa Cruz Biotechnology, Dallas, TX, United States), human anti-centromere antibody derived from human CREST patient serum (1:100, Antibodies Incorporated, California, United States), rabbit polyclonal anti-H3K9me2 antibody (1:100, Bioworld Technology Inc., St. Louis Park, MN, United States), and rabbit monoclonal anti-H3K27me3 antibody (1:100, Sangon Biotech, Shanghai, China). At least three replicates were performed, and more than 30 oocytes were observed, respectively, in the control and PQ-treated groups for each replicate.

### Mitochondrial Distribution

MitoTracker Deep Red (Invitrogen, Carlsbad, CA, United States) was used to evaluate the distribution of mitochondria in the control and PQ-treated groups. Briefly, MI oocytes were stained with pre-warmed MitoTracker Deep Red for 30 min at 37°C. After being stained with DAPI for 15 min, MI oocytes were put on glass slides for observation by a laser scanning confocal microscope (Leica TCS SP5 II, Wetzlar, Germany).

### Reactive Oxygen Species Detection and Apoptosis Assay

The Reactive Oxygen Species Assay Kit (Beyotime Institute of Biotechnology, Shanghai, China) was used to detect the level of intracellular reactive oxygen species (ROS) (Nie et al., 2020). MI oocytes were incubated with the oxidation-sensitive fluorescent probe at 37°C for 30 min and then transferred to glass slides for observation under a laser scanning confocal microscope. All the scanning parameters of the confocal system remained the same. The Annexin V-FITC Apoptosis Kit (Beyotime Institute of Biotechnology) was used to detect the level of early apoptosis. Oocytes were exposed to the acid M2 for 5 s to remove the zona pellucida and stained with 39 μl of binding buffer containing 1 μl of Annexin V FITC for 30 min in the dark. The fluorescent signal on the membrane was considered as the symbol of early apoptosis.

### Fluorescence Intensity Analysis

All pictures were captured under the same scanning parameters. Per unit area within the region of interest (ROI) of the average fluorescence intensity was examined by ImageJ software (National Institutes of Health, Bethesda, MD, United States).

### Statistical Analysis

All statistics from at least three replicated experiments were expressed as mean ± SEM and analyzed with GraphPad Prism software by one-way analysis of variance (ANOVA) analysis. A *P* value of less than 0.05 was considered as statistically significant. At least 30 oocytes were observed in the control and PQ-treated groups for each replicate.

## Results

### Paraquat Reduces the Weight of Ovaries and Disturbs First Polar Body Extrusion in Mouse Oocytes

**FIGURE 1:**
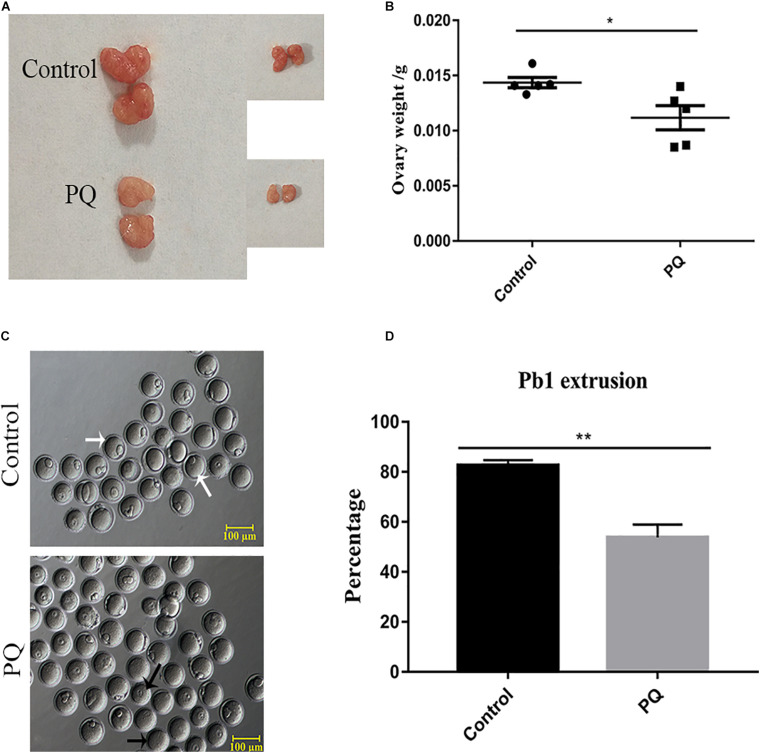
Paraquat (PQ) reduces the weight of ovary and disturbs first polar body (PB1) extrusion in mice. **(A)** Representative images of ovary from the control and PQ-treated groups. **(B)** The average weight of ovaries in the control and PQ-treated groups. **(C)** Representative images of PB1 extrusion from the control and PQ-treated groups. The white arrow indicates the oocyte with PB1 extrusion, and the black arrow indicates the oocyte without a PB1. Scale bar = 100 μm. **(D)** The percentage of PB1 extrusion in the control and PQ-treated groups. Data are presented as mean ± SEM from at least three independent experiments. ^∗^*P* < 0.05, ^∗∗^*P* < 0.01.

After PQ administration *in vivo* for 21 days, the average weight of ovaries in the PQ-treated group was significantly decreased compared with that in the control (0.01436 ± 0.00046 g, *n* = 5, control vs. 0.01118 ± 0.00110 g, *n* = 5, PQ-treated group; *P* < 0.05; Figures 1A,B). To investigate the effects of PQ on the oocyte maturation, the percentage of PB1 extrusion was directly detected. The proportion of PB1 in the PQ-treated oocytes was decreased significantly compared with that in the control oocytes (77.4 ± 3.8%, *n* = 294, control vs. 47.8 ± 3.0%, *n* = 225, PQ-treated group; *P* < 0.05; Figures 1C,D). These data demonstrated that PQ can impair the ovary and cause the meiotic arrest during oocyte maturation in mice.

### Paraquat Decreases the Embryonic Development Potential *in vivo*

**FIGURE 2:**
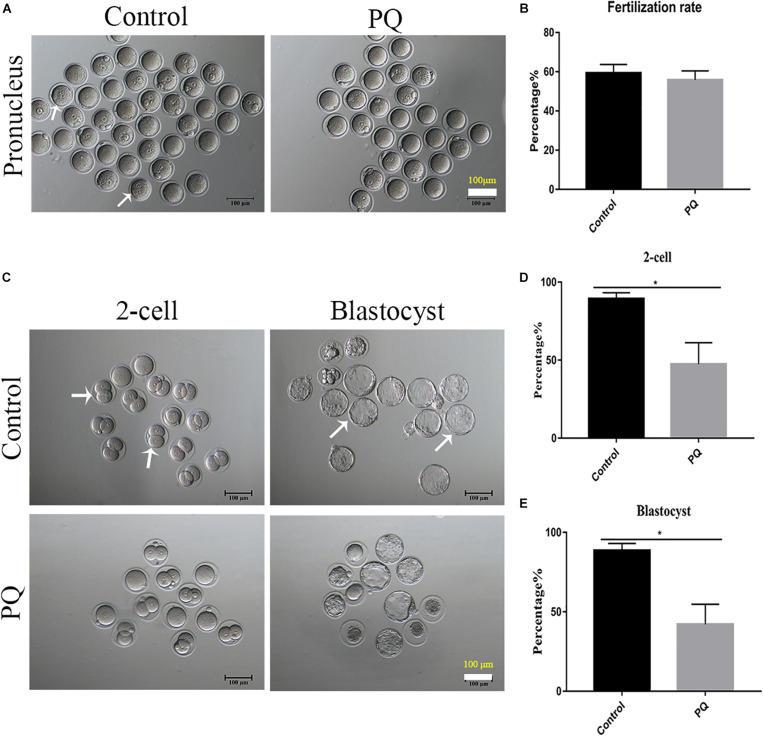
Paraquat (PQ) decreases the embryonic developmental potential in mice. **(A)** Representative images of pronuclei after *in vitro* fertilization (IVF) in the control and PQ-treated groups. The white arrow indicates the zygote with pronucleus. Scale bar = 100 μm. **(B)** The percentage of IVF rates in the control and PQ-treated groups. **(C)** Representative images of two-cell embryos and blastocysts in the control and PQ-treated groups after mating. The white arrow indicates the representative two-cell embryos and blastocysts. Scale bar = 100 μm. **(D,E)** The percentage of two-cell embryos and blastocysts in the control and PQ-treated groups after mating. Data are presented as mean ± SEM from at least three independent experiments. ^∗^*P* < 0.05.

After PQ administration *in vivo* for 21 days, the number of MII oocytes from natural estrus cycle in the PQ-treated group was significantly decreased compared with that in the control group (14.33 ± 0.7, *n* = 9, control vs. 11.56 ± 0.5, *n* = 9, PQ-treated group; *P* < 0.05). The oocyte quality directly influences the fertilization and subsequent embryo development, so the corresponding experiments were conducted. The results showed that PQ had no significant effects on the rates of IVF (59.33 ± 4.4%, *n* = 84, control vs. 55.8 ± 4.7%, *n* = 114, PQ-treated group; *P* > 0.05; Figures 2A,B). However, after mating the rates of two-cell embryos and blastocysts in the PQ-treated group were significantly lower than those in the control (two-cell embryos: 89.41 ± 3.8%, *n* = 129, control vs. 47.45 ± 13.8%, *n* = 104, PQ-treated group; blastocyst: 88.63 ± 4.5%, *n* = 129, control vs. 42.1 ± 12.7%, *n* = 104, PQ-treated group; *P* < 0.05; Figures 2C–E). These data demonstrated that PQ exposure can decrease the level of mature oocytes and impair the embryonic development potential *in vivo*, indicating that the cytoplasmic maturation is compromised.

### Paraquat Reduces the Litter Size of Female Mice and the Average Weight of Offspring

**FIGURE 3:**
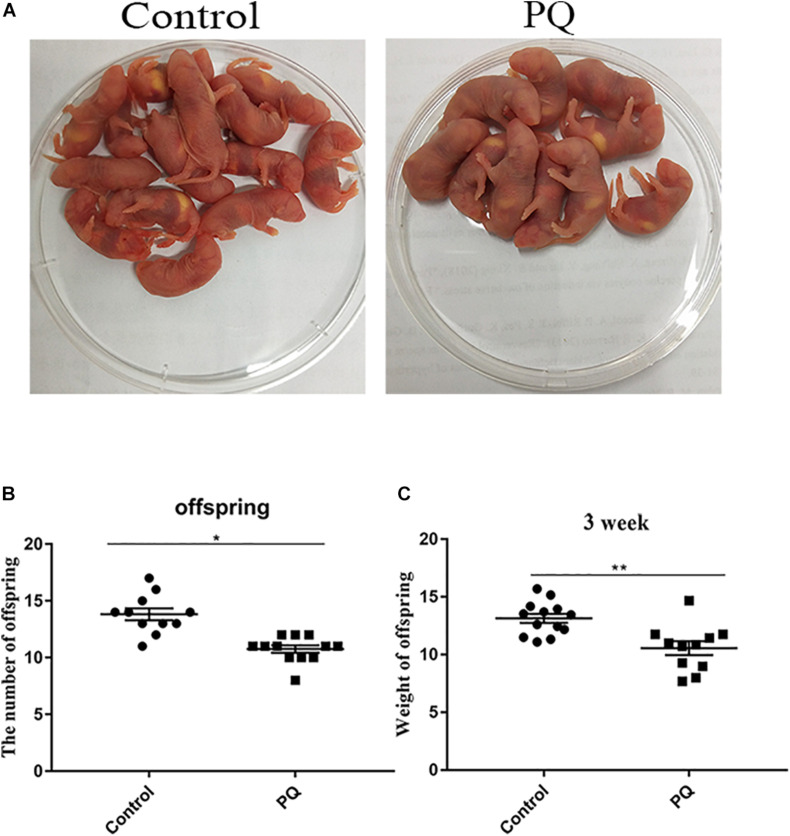
Paraquat (PQ) reduces the litter size of female mice and the weight of 3-week offspring. **(A)** Representative images of offspring in the control and PQ-treated groups. **(B)** The average numbers of litter size in the control and PQ-treated mice. **(C)** The average weight of 3-week offspring in the control and PQ-treated mice. Data are presented as mean ± SEM from at least three independent experiments. ^∗^*P* < 0.05, ^∗∗^*P* < 0.01.

Next, the numbers of offspring were examined to define the fertility. The average numbers of offspring were 14 ± 0.5, *n* = 13, control vs. 10 ± 0.4, *n* = 15, PQ-treated group; *P* < 0.05 (Figures 3A,B). Also, we examined the weight of 3-week offspring and found that the average weight in the PQ-treated group was significantly lower than that in the control. The numbers of weight were 13.2 ± 0.4 g, *n* = 13, control vs. 10.6 ± 0.6 g, *n* = 11, PQ-treated group; *P* < 0.05 (Figure 3C).

### Paraquat Affects the Meiotic Spindle and Chromosome Dynamics in Mouse Oocytes

**FIGURE 4:**
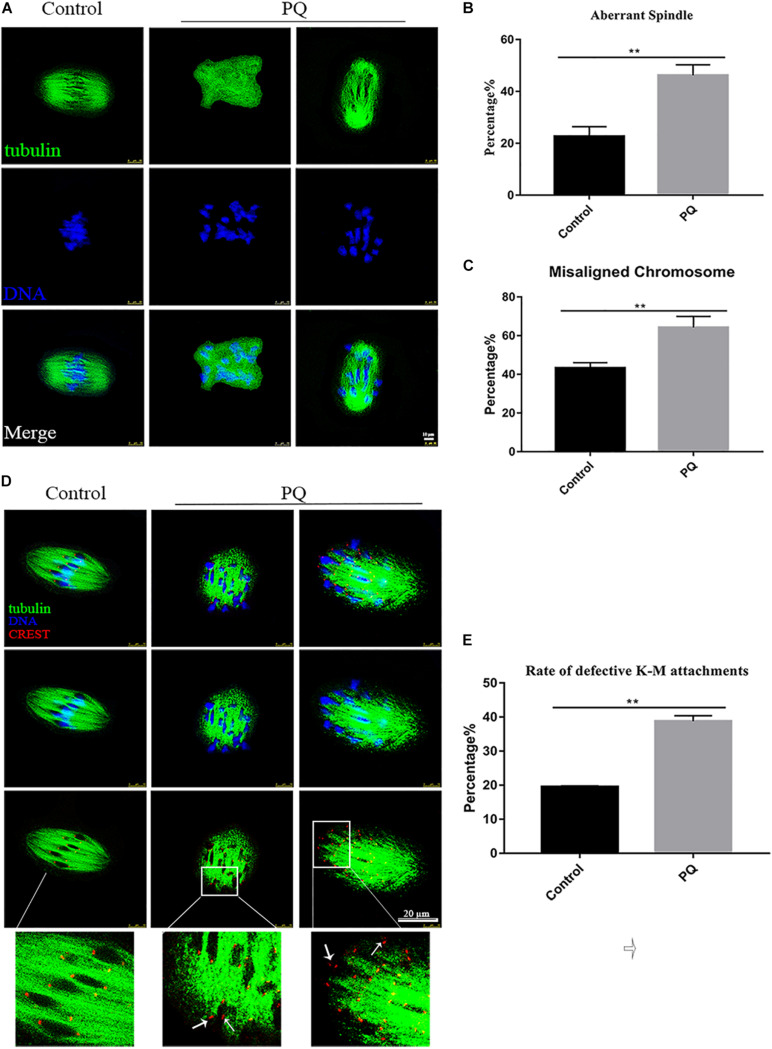
Paraquat (PQ) affects the meiotic spindle and chromosome dynamics in mouse oocytes. **(A)** Representative images of spindle morphology from the control and PQ-treated groups. Spindle (green) and DNA (blue). Scale bar = 10 μm. **(B)** The percentage of aberrant spindle in the control and PQ-treated groups. **(C)** The percentage of misaligned chromosome in the control and PQ-treated groups. **(D)** Representative images of kinetochore and microtubule (K-M) attachment from the control and PQ-treated groups. Images of kinetochores (red), microtubule (green), and DNA (blue) were observed by the confocal microscopy. The white arrow indicates the abnormal K-M attachment or no K-M attachment. Scale bar = 10 μm. **(E)** The rate of defective K-M attachments in the control and PQ-treated groups. Data are presented as mean ± SEM from at least three independent experiments. ^∗∗^*P* < 0.01.

Correct spindle assembly is one of the important factors that contribute to the faithful chromosome segregation, which ensures the formation of euploid. After treatment with PQ, the percentage of aberrant spindle in the PQ-treated group was significantly higher than that in the control group. The proportion of aberrant morphology of spindle was 22.7 ± 3.7%, *n* = 157, control vs. 44.2 ± 4.0%, *n* = 126, PQ-treated group; *P* < 0.01 (Figures 4A,B). Also, the percentage of misaligned chromosome in the PQ-treated group was significantly increased than that in the control group. The numbers were 43.3 ± 2.7%, *n* = 171, control vs. 64.2 ± 5.8%, *n* = 114, PQ-treated group; *P* < 0.01 (Figure 4C). These data indicated that PQ exposure impairs the nuclear maturation of oocyte by disrupting the meiotic spindle morphology and increasing the percentage of misaligned chromosome. And the incorrect interaction between kinetochore and microtubule (K-M) is generally considered to be associated with the abnormal spindle assembly, which could increase the percentage of aneuploidy rate in MII oocytes (Lan et al., 2018). The rate of defective K-M attachments was dramatically increased in the PQ-treated group compared with that in the control group (Figure 4D). The proportion of defective K-M attachments was 19.5 ± 0.3%, *n* = 133, control vs. 38.8 ± 1.6%, *n* = 140, PQ-treated; *P* < 0.01 (Figure 4E). These statistical data showed that PQ can impair the interaction K-M attachments during oocyte maturation.

### Paraquat Causes the Abnormal Mitochondrial Translocation in Mouse Oocytes

**FIGURE 5:**
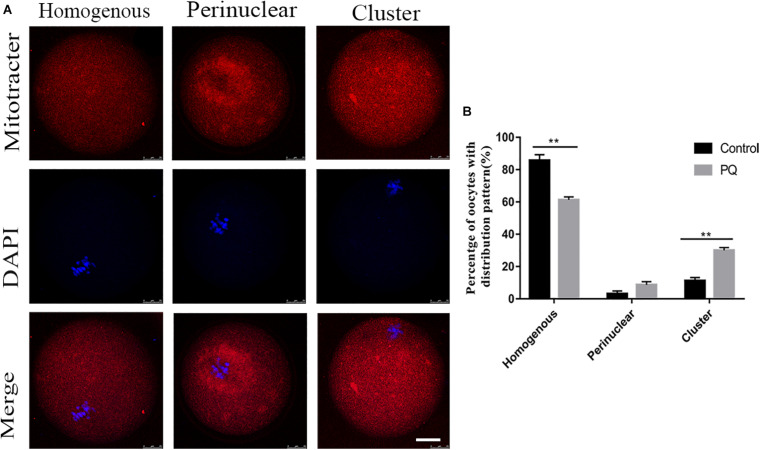
Paraquat (PQ) causes the abnormal distribution of mitochondria in mouse oocytes. **(A)** Representative images of mitochondrial distribution in the control and PQ-treated groups. Mitochondria (red) and DNA (blue). Scale bar = 25 μm. **(B)** The proportion of homogenous, perinuclear, and cluster mitochondrial distribution in the control and PQ-treated groups. Data are presented as mean ± SEM from at least three independent experiments. ^∗∗^*P* < 0.01.

Mitochondria could be moved to the specific regions by microtubule and associated with the concentrating ATP or calcium during oocyte maturation and fertility. So we used MitoTracker Deep Red to detect the distribution of mitochondria after treatment with PQ. The percentage of homogenous distribution of mitochondria was remarkably decreased, and the number of cluster distribution of mitochondria was significantly increased in the PQ-treated group, compared with that in the control group (Figure 5A). The rates of homogenous distribution were 85.7 ± 3.6%, *n* = 133, control vs. 61.3 ± 1.8%, *n* = 140, PQ-treated group, *P* < 0.01; the rates of perinuclear distribution were 3.1 ± 1.8%, *n* = 133, control vs. 8.7 ± 1.9%, *n* = 140, PQ-treated group, *P* > 0.05; and the rates of cluster distribution was 11.22 ± 3.4%, *n* = 133, control vs. 30.0 ± 1.7%, *n* = 140, PQ-treated group, *P* < 0.01 (Figure 5B). These data revealed that PQ exposure can affect the translocation of mitochondria from perinuclear to cytoplasm.

### Paraquat Increases the Level of Intracellular Reactive Oxygen Species and Induces the Early Apoptosis in Mouse Oocytes

**FIGURE 6:**
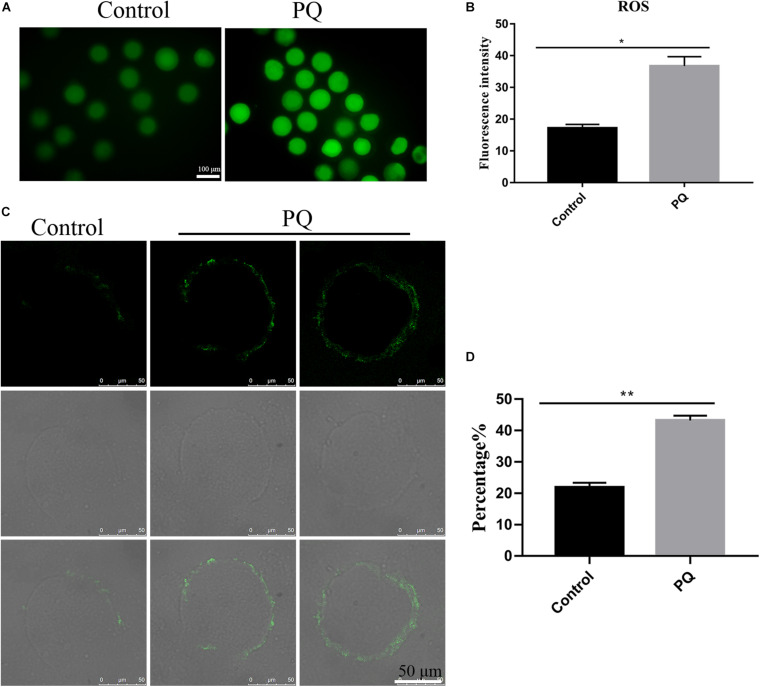
Paraquat (PQ) increases the level of intracellular reactive oxygen species (ROS) and induces the apoptosis in mouse oocytes. **(A)** Representative images of ROS (green) in the control and PQ-treated groups. Scale bar = 100 μm. **(B)** The fluorescence intensities of ROS in the control and PQ-treated groups. **(C)** Representative images of early apoptosis in the control and PQ-treated groups. Oocytes with the Annexin V signals (green) at membrane were considered as the early apoptosis. Scale bar = 50 μm. **(D)** The percentage of early apoptosis in the control and PQ-treated groups. Data are presented as mean ± SEM from at least three independent experiments. ^∗^*P* < 0.05; ^∗∗^*P* < 0.01.

Mitochondrial dysfunction induces a high level of intracellular ROS to damage the quality of oocytes (Giorgi et al., 2018), so we tested the level of ROS with Reactive Oxygen Species Assay Kit by fluorescent analysis. The ROS level was visibly increased and the fluorescent signal was significantly higher in the PQ-treated oocytes, compared with the control (Figure 6A). The fluorescence intensity of ROI was 17.2 ± 1.2%, *n* = 140, control vs. 36.7 ± 2.9%, *n* = 149, PQ-treated group; *P* < 0.01 (Figure 6B). These data suggested that PQ exposure increases the level of intracellular ROS in mouse oocytes.

Since a too high level of intracellular ROS could induce apoptosis (Tiwari et al., 2015), next, we evaluated the level of apoptosis by detecting Annexin V signals in oocytes. The fluorescent signal on the membrane in the control group was significantly lower than that in the PQ-treated groups (Figure 6C). The percentages of early apoptosis were 21.87 ± 1.5%, *n* = 141, control vs. 43.2 ± 1.5%, *n* = 169, PQ-treated group; *P* < 0.01 (Figure 6D). All these results indicated that PQ exposure can induce the early apoptosis in mouse oocytes.

### Paraquat Reduces the Levels of Histone Methylation in Mouse Oocytes

**FIGURE 7:**
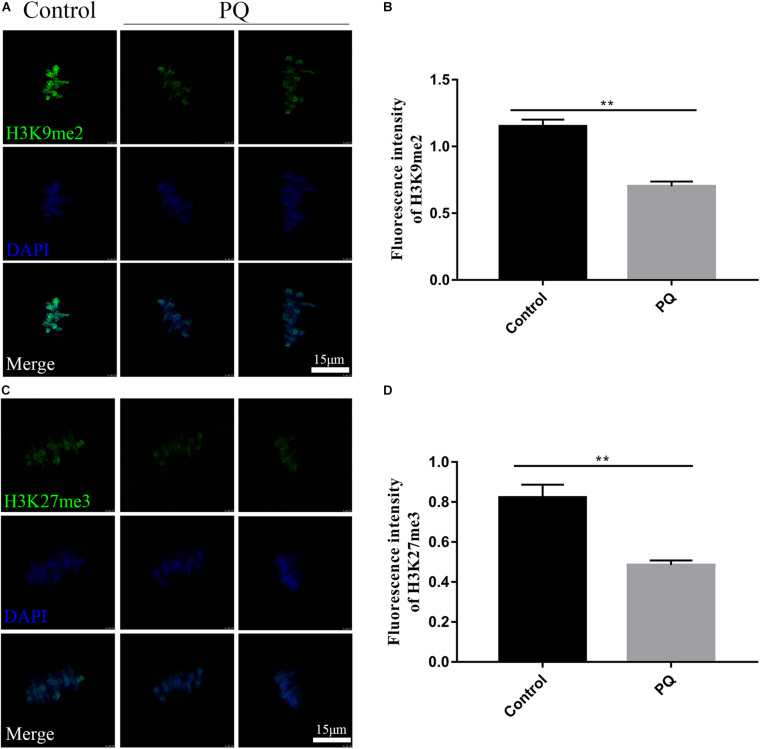
Paraquat (PQ) reduces the levels of histone methylation in mouse oocytes. **(A)** Representative images of H3K9me2 (green) and DNA (blue) in the control and PQ-treated groups. Scale bar = 15 μm. **(B)** The fluorescence intensities of H3K9me2 in the control and PQ-treated groups. **(C)** Representative images of H3K27me3 (green) and DNA (blue) in the control and PQ-treated groups. Scale bar = 15 μm. **(D)** The fluorescence intensities of H3K27me3 in the control and PQ-treated groups. Data are presented as mean ± SEM from at least three independent experiments. ^∗∗^*P* < 0.01.

The correct epigenetic modification is a crucial element for successful fertilization and zygote cleavage (Kim et al., 2019). Next, we investigated the effects of PQ on histone methylation of oocytes. Compared with the control group, the fluorescence intensity of H3K9me2 was observably reduced in the PQ-treated oocytes (Figure 7A). The fluorescence intensities of H3K9me2 were 1.2 ± 0.05, *n* = 95, control vs. 0.7 ± 0.04, *n* = 96, PQ-treated group; *P* < 0.01 (Figure 7B). The fluorescence intensity of H3K27me3 in the PQ-treated oocytes was lower than that in the control oocytes (Figure 7C). The number of fluorescence intensities of H3K27me3 was 0.8 ± 0.06, *n* = 97, control vs. 0.5 ± 0.02, *n* = 93, PQ-treated group; *P* < 0.01 (Figure 7D). All these data demonstrated that PQ exposure can decrease the levels of histone methylation in mouse oocytes.

## Discussion

Paraquat is detected in pregnant woman urine, and the PQ concentration in urinary increases dramatically by contact with certain agricultural activities. It is reported that 55% of newborn meconium samples contains measurable PQ (Konthonbut et al., 2018). PQ on the female fertility deserves more attention. In this study, we elucidated the effects of PQ on mouse oocytes and offspring from the following aspects: oocyte maturation; rates of fertilization and early embryo development; litter size; spindle morphologies and chromosome alignment; K-M attachments; mitochondrion-related functions including redistribution, oxidative stress, and apoptosis; and epigenetic modifications. All these combined data indicate that PQ exposure impairs the oocyte quality to damage female fertility via affecting the nuclear and cytoplastic maturation of oocytes.

The quality of oocytes is associated with oocyte maturation, viability, fertilization, and the embryo development, etc. Meiotic maturation of oocytes can be divided into nuclear maturation and cytoplasmic maturation. The rate of PB1 extrusion is significantly decreased in the PQ-treated group, which indicates that the nuclear maturation is affected. In addition, PQ exposure decreases the rates of two-cell embryo and blastocyst, indicating that some oocytes may undergo the nuclear maturation but do not accomplish the total cytoplasmic maturation. The numbers of litter size and the weight of 3-week offspring are dramatically reduced in the PQ-treated mice, further verifying that PQ impacts the developmental competence of oocytes caused from the failure of cytoplasmic maturation. All these data demonstrate that PQ exposure can reduce the female fertility by impairing the oocyte quality via affecting both nuclear and cytoplasmic maturation.

Meiotic arrest is usually caused by the abnormal spindle assembly and chromosome alignment. Spindle characteristics are involved in fertilization and embryonic development (Cooke et al., 2003; Shen et al., 2006), and aberrant spindles are considered crucial for increasing the fetal miscarriage, infertility incidence, and abnormal conceptions, which result in birth defects (Battaglia et al., 1996). Faithful chromosome segregation in the oocytes with nuclear maturation requests the correct connection between the kinetochore and spindle microtubule to help the bi-orientation of homologous chromosome. PQ exposure caused the aberrant K-M attachment and spindle-chromosome assembly, which leads to the distinct nuclear maturation defects.

Deficient or abnormal cytoplasmic maturation of oocyte is considered to damage the subsequent embryo development. Notably, the cytoplasmic maturation is more complicated. It includes many factors, for example, organelle preparation and redistribution (Marteil et al., 2009). Mitochondria are the most important organelles, which produce energy and play other necessary functions. Originally, mitochondria are aggregated in the perinuclear area of oocytes. With the mature procession, mitochondria shift to the inner cytoplasm to play their roles in the mature oocytes (Sun et al., 2001). PQ administration inhibits the translocation of mitochondria from the perinuclear area and then affects the cytoplasmic maturation, which subsequently influence the early embryo development. Meanwhile, a previous study has shown that mitochondria are the center of oxidative metabolism and principal site of ROS production (Giorgi et al., 2018). Mitochondrial dysfunction caused by PQ elevates the ROS production, which is supposed to be a factor to trigger apoptosis (Bokov et al., 2004; Elmore, 2007). Therefore, it is not surprising that the levels of intracellular ROS and early apoptosis were detected in the PQ-treated oocytes. All of the combined data indicate that PQ affects the cytoplasmic maturation by impairing the mitochondrial redistribution and inducing apoptosis through accumulation of ROS.

Epigenetic modifications are involved in the changes of phenotype without altering the sequence of DNA. Histone modifications play crucial roles in sensing, processing, and repairing damaged DNA to ensure the cellular homeostasis and the integrity of genetic information (Kim et al., 2019). The main changes of histone modifications include methylation, acetylation, and phosphorylation. Histone modifications can result in the activation or suppression of gene expression to impact the development of oocytes and the embryonic development potential (Crea et al., 2014; Nowacka-Zawisza and Wisnik, 2017). It has been reported that histone H3 lysine on lysine 9 (H3K9) methylation is crucial for heterochromatin formation (Audergon et al., 2015), and histone H3 on lysine 27 (H3K27) methylation is a marker of transcriptional repression and frequently found in the heterochromatin (Pan et al., 2018). After treatment with PQ, the fluorescence intensity of H3K9me2 and H3K27me3 is distinctly reduced, suggesting that PQ changes the chromatin configuration in mouse oocytes. These data imply that PQ may deteriorate the embryonic development potential due to affecting the transcriptional activity of oocytes.

In conclusion, our research demonstrates that PQ exposure can impair the oocyte quality to weaken the female fertility by impairing the nuclear and cytoplasmic maturation during oocyte meiosis in mice.

## Data Availability Statement

The original contributions presented in the study are included in the article/supplementary material, further inquiries can be directed to the corresponding authors.

## Ethics Statement

The animal study was reviewed and approved by the Animal Research Committee of Qingdao Agricultural University.

## Author Contributions

Y-LS and SY conceived and designed the experiments. Y-LS performed the experiments. Y-LS, L-LY, Z-JG, and SY analyzed the data and participated in discussion. Q-YS, SY, YZ, WS, and S-ML contributed reagents, materials, and analysis tools. Y-LS, SY, and X-LW wrote and revised the manuscript. All authors contributed to the article and approved the submitted version.

## Conflict of Interest

The authors declare that the research was conducted in the absence of any commercial or financial relationships that could be construed as a potential conflict of interest.
